# New Model Systems and the Development of Targeted Therapies for the Treatment of Neurofibromatosis Type 1-Associated Malignant Peripheral Nerve Sheath Tumors

**DOI:** 10.3390/genes11050477

**Published:** 2020-04-28

**Authors:** Kyle B. Williams, David A. Largaespada

**Affiliations:** 1 Department of Pediatrics, University of Minnesota, Minneapolis, MN 55455, USA; 2 Masonic Cancer Center, University of Minnesota, Minneapolis, MN 55455, USA

**Keywords:** malignant peripheral nerve sheath tumors, plexiform neurofibromas, Schwann cells, neurofibromatosis type 1 syndrome, neurofibromin 1, genetically engineered mouse models

## Abstract

Neurofibromatosis Type 1 (NF1) is a common genetic disorder and cancer predisposition syndrome (1:3000 births) caused by mutations in the tumor suppressor gene *NF1*. *NF1* encodes neurofibromin, a negative regulator of the Ras signaling pathway. Individuals with NF1 often develop benign tumors of the peripheral nervous system (neurofibromas), originating from the Schwann cell linage, some of which progress further to malignant peripheral nerve sheath tumors (MPNSTs). Treatment options for neurofibromas and MPNSTs are extremely limited, relying largely on surgical resection and cytotoxic chemotherapy. Identification of novel therapeutic targets in both benign neurofibromas and MPNSTs is critical for improved patient outcomes and quality of life. Recent clinical trials conducted in patients with NF1 for the treatment of symptomatic plexiform neurofibromas using inhibitors of the mitogen-activated protein kinase (MEK) have shown very promising results. However, MEK inhibitors do not work in all patients and have significant side effects. In addition, preliminary evidence suggests single agent use of MEK inhibitors for MPNST treatment will fail. Here, we describe the preclinical efforts that led to the identification of MEK inhibitors as promising therapeutics for the treatment of NF1-related neoplasia and possible reasons they lack single agent efficacy in the treatment of MPNSTs. In addition, we describe work to find targets other than MEK for treatment of MPNST. These have come from studies of RAS biochemistry, in vitro drug screening, forward genetic screens for Schwann cell tumors, and synthetic lethal screens in cells with oncogenic *RAS* gene mutations. Lastly, we discuss new approaches to exploit drug screening and synthetic lethality with *NF1* loss of function mutations in human Schwann cells using CRISPR/Cas9 technology.

## 1. Neurofibromatosis Type 1 Syndrome and Associated Peripheral Nerve Sheath Tumors

Neurofibromatosis type 1 (NF1) syndrome is a common, autosomal dominant genetic disease that causes a predisposition to several kinds of tumors, especially a spectrum of benign and malignant forms of peripheral nerve sheath tumors (PNSTs). Patients with NF1 have inherited one mutant copy of the *NF1* gene, encoding the Ras GTPase activating protein neurofibromin, and tumors develop after somatic cell loss of the remaining wild type *NF1* allele. Benign Schwann cell PNSTs in patients with NF1 called plexiform neurofibromas (PNs) are common and problematic, occurring in roughly 60% of patients [1]. PNs have limited treatment options and can cause significant pain and morbidity. These PNs are composed of a complex mixture of cell types, but the neoplastic component is derived from a Schwann cell lineage cell, which has undergone loss of heterozygosity (LOH) of the *NF1* locus, with retention of the mutant allele [2]. Thus, these PN cells have no functional copies of *NF1* and do not produce any functional neurofibromin protein. Other reactive cell types within the PN, several of which are thought to help initiate and drive PN growth, include perineural and CD34+ fibroblasts, endothelial cells, neurons, and various cells of hematopoietic origin including mast cells, macrophages, and T cells [2,3,4]. PNs can affect any peripheral nerve, are thought to be congenital, and often grow aggressively during childhood [3]. A feared complication of the PNs is malignant transformation.

A newly recognized type of tumor along the spectrum of neurofibroma to malignant peripheral nerve sheath tumors (MPNST) is called atypical neurofibromatosis neoplasms of uncertain biological potential (ANNUBP) [5]. ANNUBP have at least two of three features not common in PNs, including loss of neurofibroma architecture, high cellularity, and high mitotic activity [5,6]. ANNUBPs are very important because they may well be premalignant tumors and an important transition step to MPNST. They often show loss of nuclear p16INK4A protein expression with variable loss of S100 and SOX10 expression, which are also common findings in MPNSTs [5]. ANNUBP have frequent *CDKN2A/CDKN2B* gene copy number loss [6,7].

MPNSTs are aggressive soft tissue sarcomas thought to be derived from PN Schwann cells. MPNSTs can occur in any nerve and do not respond to current therapies. In fact, MPNSTs are the most common cause of death of patients with NF1 [1]. It is estimated that roughly half of all MPNST patients have NF1, the other half of MPNSTs occur sporadically in patients without any obvious cancer predisposition syndrome [8]. As might be expected, sporadic MPNST occurs more commonly in older patients compared to patients with NF1 syndrome, many of whom develop MPNSTs in adolescence or young adulthood. While disputed, some data suggests that MPNSTs developing in the context of NF1 syndrome have worse clinical outcomes [9].

## 2. Molecular Genetics of the *NF1* Gene Product and MPNST

As mentioned above, *NF1* encodes a large GTPase activating protein (GAP) called neurofibromin. GAPs increase the intrinsic GTPase activity of small GTPases, such as the Ras superfamily of proteins. Neurofibromin has GAP activity for several Ras proteins including HRAS, KRAS, NRAS, RRAS, and perhaps others [10]. Patients with NF1 are heterozygous for *NF1* gene mutations, but the benign and malignant tumors that develop in these patients are caused, in part, by somatic cell loss of the remaining wildtype *NF1* allele [11]. Preclinical models suggest, many NF1-associated tumors have increased and prolonged RAS activation and MEK/ERK signaling after stimulation [1,12]. However, monotherapy often leads to emergence of drug resistance, and work from our lab indicates MEK inhibition can synergize with other therapeutics, such as mTOR inhibitors [13]. It is unclear if MEK inhibition will be useful in MPNST treatment, but preclinical data suggests that it may [12]. It is likely, however, that other recurrent genetic changes in MPNSTs make them relatively resistant to treatment with a MEK inhibitor alone [14,15,16,17]. Moreover, these other genetic abnormalities may be associated with additional drug sensitivities, which might be usefully exploited along with MEK inhibition. 

MPNSTs also have frequent loss of either *CDKN2A* or *TP53* [18,19,20]. In addition, loss of function mutations in the Polycomb repressive complex 2 (PRC2) component genes, such as *SUZ12* or *EED*, are now known to be *very* common in MPNSTs, occurring in ~70% of NF1-associated cases and more than 90% of sporadic cases [21,22]. PRC2 represses many loci by causing histone H3 lysine 27 trimethylation (H3K27me3) in promoter regions [23]. Loss of H3K27me3, known to be caused by mutations in *SUZ12* or *EED*, is an indicator of a bad prognosis in MPNST [24]. These common mutations in chromatin remodeling machinery provide unique avenues of therapeutic targeting. In fact, it has been reported that *SUZ12* mutant MPNSTs are sensitive to bromodomain inhibitors, such as JQ1 [25]. To determine if such therapeutic ideas might have merit in the clinic, it is critical to ensure that the very best model systems for MPNSTs are utilized.

## 3. MPNST Model Systems

Human MPNST tumor models for testing new therapeutic ideas include genetically engineered mouse models (GEMMs), genetically engineered zebrafish [26,27,28,29], and established human MPNST cell lines [30]. There are two main problems with these approaches. The most often used GEMM does not recapitulate all of the genetic features of human MPNSTs, especially NF1 syndrome-associated MPNSTs, which likely develop from a pre-existing PN or ANNUBP. Secondly, there are relatively few established human MPNST cell lines in common usage in the field. We believe that MPNST therapy would benefit from development of better model systems. These should include: (1) better GEMMs, (2) genetically engineered human cells of the correct lineage(s) for an MPNST, and (3) use of primary human MPNSTs grown in vitro and in vivo as patient derived xenografts. Below, each of these three types of models are described in more detail including work already done and published, current efforts, and future innovations.

### 3.1. Genetically Engineered Mouse Models (GEMM)

Several GEMMs of MPNSTs have been published. While the most relevant incorporate loss of the *Nf1* gene, others have shown that Ras oncogene activation itself can cooperate with *Pten* loss to induce MPNST-like tumors [31]. Others have shown that overexpression of certain growth factor ligands or receptors in Schwann lineage cells can also induce these tumors [32]. These models have been useful for many things, including testing new therapies. However, major current limitations of these models include: few demonstrate metastasis (as is common in human MPNSTs), most do not incorporate loss of PRC2 function, and it is currently unclear if any really represent human MPNSTs at the transcriptomic level or reflect human MPNST molecular subtypes if they exist.

Several investigators have shown that Schwann cell lineage overexpression of growth factor receptors or ligands can promote peripheral nerve sheath tumor development. The ligand neuregulin-1, which binds to the ERBB3 and ERBB4 receptors, when expressed from the P_0_ promoter leads to peripheral nerve sheath tumor formation [33,34], which is accelerated to a higher grade by concomitant loss of *Trp53* [35]. Expression of human epidermal growth factor receptor (hEGFR) from a Desert hedgehog (*Dhh*) promoter can induce neurofibromas by itself [36], and cooperate with expression of a dominantly acting *Trp53^R270H^* mutant [37], or loss of one or two copies of *Pten* [32]. Human *PTEN* is often deleted in human MPNSTs and is expressed at reduced levels compared to normal human Schwann cells or Schwann cells from benign neurofibromas [38]. Indeed, codeletion of *Nf1* and *Pten* in *Dhh-Cre* positive cells caused rapid high grade peripheral nerve sheath tumors [38]. Another paper also showed that loss of *Pten* potently accelerated high grade MPNST-like tumors in the presence of Schwann cell lineage expression of *Kras^G12D^* [31]. Taken together these data strongly suggest that *PTEN*-regulated pathways are major suppressors of progression to MPNST. Which PTEN-regulated processes are most important is not clear, but evidence suggests AKT-driven beta-catenin activation [39], and/or mTOR activation might be critical effectors [40]. *CDKN2A* is a well-established human MPNST tumor suppressor, frequently lost in pre-malignant, atypical neurofibromas [5,41]. When combined with deletion of *Nf1* in the Schwann cell lineage, signatures of senescence are suppressed in resultant neurofibromas, which appear as faster developing, higher grade tumors than produced by deletion of *Nf1* alone [7]. Thus, this work provides a good model of ANNUBP and it suggests that senescence limits neurofibroma progression. Therefore, therapies that target senescent cells, so called senolytics [42], could be useful to prevent peripheral nerve sheath tumor progression. 

Two papers report *Sleeping Beauty* (SB) transposon-based insertion mutation screens in *Dhh-Cre* or *Cnp-Cre* positive cells in mice in vivo [43,44]. These studies were done on an *Nf1^flox/flox^* [44] or *loxP-STOP-loxP-Trp53^R270H^* (*LSL-Trp53^R270H^*) plus *Cnp-hEGFR* mutant backgrounds [43]. The result of SB mutagenesis in *Dhh-Cre* positive cells on the *Nf1^flox/flox^* background was to increase tumor multiplicity but not tumor grade, which were all benign neurofibromas. However, SB mutagenesis in *Cnp-Cre* positive cells on a *LSL-Trp53^R270H^* plus *Cnp-hEGFR* background resulted in accelerated high-grade MPNSTs like tumor development in most mice. While this model is primarily useful for the genes and pathways it revealed as potential drivers of MPNST, tumors made in this way could be used to select for additional tumor phenotypes because the tumors can be allografted and SB transposon mutagenesis is an ongoing process. It may be possible to select for metastatic potential and treatment resistance using this SB model. In any case, the SB screen revealed PTEN regulated signaling (e.g., PI3 kinase), Wnt-beta catenin signaling, RAS-MAPK signaling, Hippo/Yap signaling, Myc activation through FOXR2, SHH signaling, and other pathways as potential drivers of MPNST [43]. Moreover, while inactivating SB transposon insertion mutations in *Eed* and *Suz12* were not recovered in the screen, inactivating *Jarid2* and *Nsd1* insertion mutations were recovered [43]. JARID2 is a critical component of the PCR2 complex required for localization of the PRC2 complex on chromatin, and has been reviewed before [45]. NSD1 is required to prevent histone H3 lysine 27 trimethylation (H3K27me3) from spreading to new regions of chromatin genome-wide, the effect of which is to decrease H3K27me3 in its usual domains [46]. Thus, the SB screen did reveal a role of H3K27me3 function in MPNST suppression.

Perhaps the most well-studied GEMM for MPNST is the so-called *NPCis* mouse, in which loss of function mutations in *Nf1* and *Trp53* are placed in a Cis configuration as both genes reside closely linked on chromosome 11 [47,48]. As in the mouse, these two TSGs are closely linked in the human genome. In *NPCis* mice, therefore, a single loss of heterozygosity event can result in complete elimination of both TSGs. Two different groups reported that *NPCis* mice develop a variety of sarcomas, including MPNST-like tumors, as well as gliomas [48]. Strain-specific effects influence the frequency of MPNST-like tumors, with more developing on a 129/SvJ background than on a C57BL/6J background [49]. The *NPCis* mouse model has been used to test a variety of new therapies [40,50,51,52,53]. The major limitation of the *NPCis* model may have to do with the fact that these tumors do not develop through a process that involves progression from benign neurofibroma (with loss of *Nf1* only), to pre-malignant atypical neurofibroma (with loss of *Nf1* and *Cdkn2a*), to MPNST with loss of *Suz12* or *Eed*. However, heterozygous germline loss of function mutations in *Nf1* and *Suz12* have been combined in Cis on chromosome 11 and this resulted in the acceleration of neurofibroma and MPNST-like tumors [25]. Furthermore, heterozygous germline mutations in *Nf1, Trp53* and *Suz12* have been combined and the result was acceleration of MPNST-like tumor formation, along with other tumor types including glioma, lymphoma and histiocytic sarcoma [25]. The utility of the MPNST models utilizing the *Suz12* germline mutation is limited by the fact that other types of cancer develop in the mice, the highly stochastic nature of tumor development, and lack of a suitable method for labeling the MPNST cells as they develop so that in vivo imaging might be accomplished. Some of these concerns could be addressed using conditional alleles for *Cdkn2a* or *Trp53* and for *Suz12*. Another approach would be to use somatic cell editing in situ. Indeed, adenoviral delivery of Cre to the sciatic nerves of *Nf1^flox/flox^; Cdkn2a^flox/flox^* mice resulted in development of MPNST-like tumors at the injection site [54]. This model was used to demonstrate a positive, driver role for the *Nf1+/−* heterozygous field in promoting MPNST-like tumor development [55]. More recently, it has been shown that adenovirus-mediated somatic cell delivery of single guide RNAs (sgRNA) and Cas9 to the sciatic nerve can produce MPNST-like tumors [56]. The flexibility of this approach is promising since building murine MPNST-like tumors that are truly genetically similar to their human counterparts may require layering many mutations in the correct temporal order. Achieving this remains a major challenge in the field.

### 3.2. Human MPNST Cell Lines and Patient Derived Xenografts

The MPNST research field has been hampered by a relative dearth of established cell lines [57,58]. Moreover, cell lines that do exist have not been well characterized. Commonly, only a few are used to test new hypotheses. Critically, none are included in large scale whole exome/whole genome sequencing, gene expression profiling, drug, RNAi, or CRISPR/Cas9 screening projects. The field will benefit tremendously from better characterization of existing lines and establishment of more human MPNST cell lines. Similarly, only a few human MPNST patient derived xenografts (PDXs) have been described in the literature [59,60]. Apparently, MPNST PDXs can be fairly readily established in immunodeficient mice and this also has been our experience using NOD-*Rag1^null.^ Il2rg^null^* (NRG) mice. New efforts to make additional human MPNST cell lines and PDXs are underway and some of these new these resources are available to the research field already (https://www.hopkinsmedicine.org/kimmel_cancer_center/centers/pediatric_oncology/research_and_clinical_trials/pratilas/nf1_biospecimen_repository.html).

### 3.3. Human Cell-Based Models for MPNST

In theory, engineering relevant mutations into primary human cells of the correct lineage would provide models useful for synthetic lethal genetic screens and drug screens. Models of neurofibroma and MPNST made in this way would also guard against the possibility that mouse cells fail to accurately recapitulate aspects of human cell biology relevant to MPNST development. Making a permanent, non-perishable cell line resource of this kind is difficult using purified, primary human Schwann cells as they have limited proliferative potential in vitro and are difficult to culture without contaminating fibroblasts [61]. For this reason, Dr. Margaret (Peggy) Wallace, Ph.D., at the University of Florida, Gainesville, has pioneered the generation of immortalized human Schwann cells from normal nerves of non-patients with NF1 and patients with NF1, as well as neurofibromas [62]. These cells were immortalized using lentiviral transduction of human *TERT* and murine *Cdk4* transgenes. These immortalized cells have been useful for testing the effects of candidate MPNST oncogenes and tumor suppressor genes on human Schwan transformation [43,63]. The *NF1−/−* plexiform neurofibroma derived Schwann cells provide a useful cell culture model of these benign tumors [62], and provide a logical platform for studying progression to MPNST.

A permanent, non-perishable, and human cell-based model for MPNST modeling would be best accomplished using induced pluripotent stem cells (iPSC). Indeed, *NF1−/−* iPSC have been made from plexiform neurofibroma cells from patients with NF1 [64]. When these *NF1−/−* iPSCs were differentiated to neural crest cells and subsequently Schwann cells, it was found that they had an enhanced proliferation rate, poor myelination ability, and a tendency to form 3D spheres like those from primary neurofibromas compared to Schwann cells made from wild type iPSC. Thus, these Schwann cells made from a *NF1−/−* iPSC represent a valuable model to study and treat plexiform neurofibromas. It remains to be seen whether these iPSC, or differentiated progeny derived from them, can be used to model ANNUBP and MPNST by knocking out *CDKN2A* and *SUZ12*. It is also unclear how the differentiation state of the target cell and order of mutation would affect the outcome of studies like these. In any case, CRISPR/Cas9 based models produced this way are an ideal method to produce isogenic sets of relevant human cells for drug and synthetic genetic lethality screens. 

### 3.4. Synthetic Lethality as A Tool for NF1 Drug Discovery

**Figure 1:**
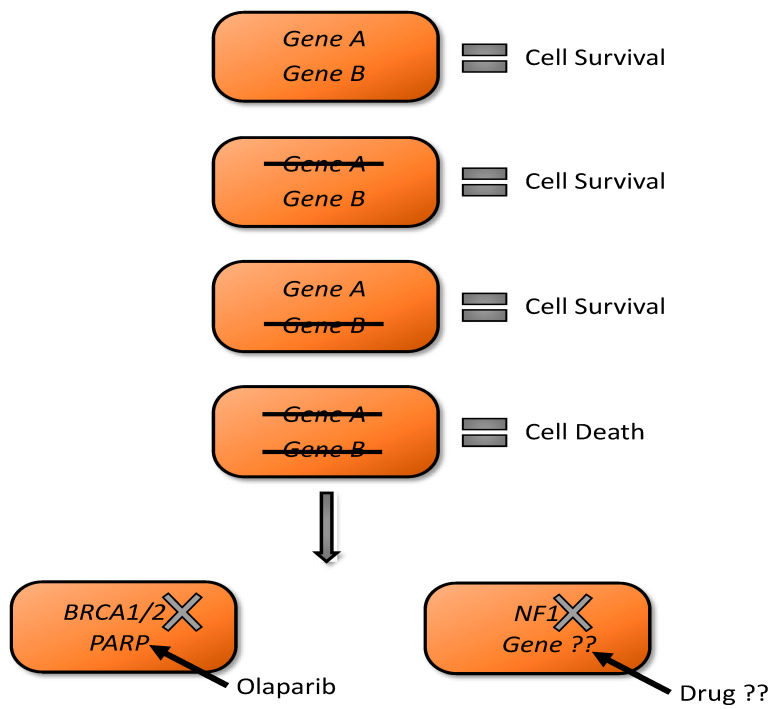
Synthetic lethality if the genetic incompatibility of the loss of two independent gene products results in cell death. Whereas a hypothetical cell can survive with *either* loss of *Gene A* or *Gene B*, it cannot live with loss of both simultaneously. A prime example of this being exploited therapeutically is in *BRCA1/2* mutated cancers and their sensitivity to PARI inhibitors such as olaprarib. Similarly, loss of *NF1* could render human Schwann cells sensitive to pharmacologic intervention with novel agents.

Synthetic lethality is the genetic incompatibility of the loss of two or more gene products, leading to cell death. However, the deficiency of any of these gene products on their own results in viable cells (Figure 1). The notion of synthetic lethality was first described in work on Drosophila genetics in the 1920′s by Calvin B. Bridges and others who noticed parental lines of flies harboring mutations were able to successfully reproduce, yet when these independent mutant lines were crossed it was not possible to obtain viable offspring with a combination phenotype [65]. Using the power of synthetic lethality to identify novel vulnerabilities in a given cancer based on defined genetic backgrounds was first suggested over 20 years ago [66]. The first type of investigation to move to clinical relevance was that of the use of inhibitors of poly(ADP-ribose) polymerase (PARP) to selectively kill BRCA-2 deficient tumors [67]. Now PARP inhibitors such are olaparib are approved for BRACA mutated ovarian cancers [68].

Treatment options for plexiform neurofibromas and malignant peripheral nerve sheath tumors (MPNST) are limited, relying mostly on surgical resection and broad-spectrum chemotherapy. The genetic basis of NF1 syndrome is well suited for using synthetic lethal genetic screens and related approaches to uncover unique variabilities in *NF1* deficient cells, as well as cells closely mimicking the genetics of an MPNST. Loss of the *NF1* gene could uncover sensitivity to loss or impairment of another gene or pathway. All neurofibromas, and derived MPNSTs, harbor *NF1*-deficient Schwann cells, which may have re-wired signaling to reveal unique vulnerabilities. However, the synthetic lethal interactions after loss of *NF1* are likely to be highly context dependent. Ideally, they should be operative in human Schwann cells. The discovery of such interactions would most easily be found using pairs of isogenic, human *NF1*-deficient and *NF1*-proficient Schwann cells. Genome engineering technologies, such as CRISPR/Cas9, allow introduction of clinically relevant mutations into cells of the correct tissue type for the disease being studied. Cells engineered in this manner would be poised to be used for novel drug discovery for not only NF1-associated malignancies, but many other cancers harboring loss of tumor suppressors or with known oncogenic drivers [69]. 

**Figure 2:**
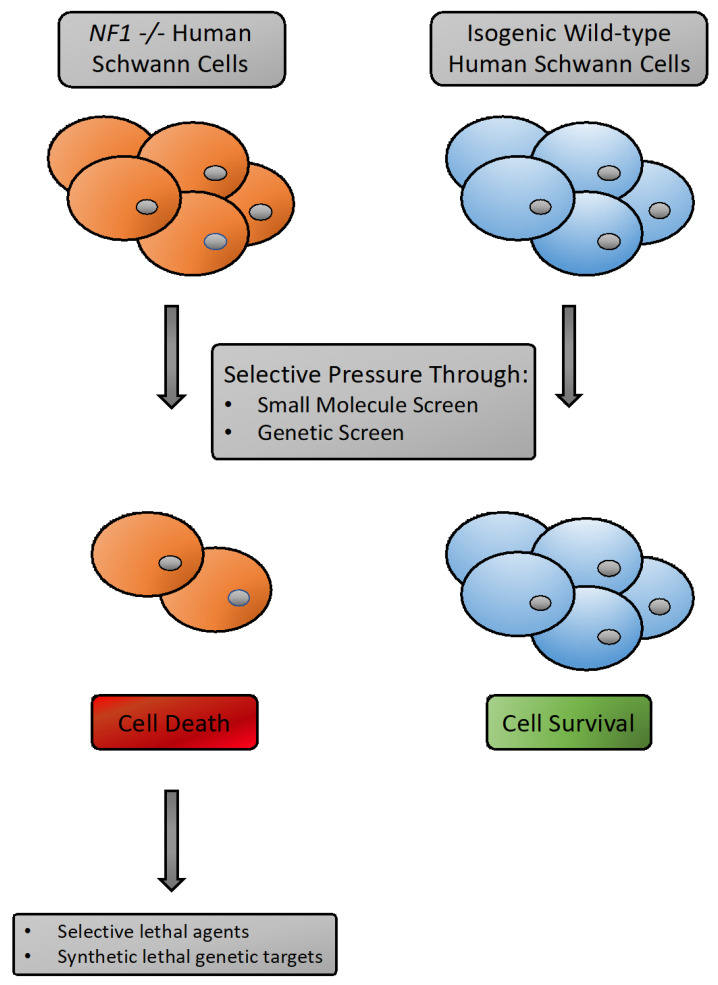
Screening scheme utilizing *NF1-*deficeint immortalized or iPSC derived human Schwann cells. *NF1 −/−* cells (of either origin) and their isogenic match parental lines can be used in selective lethal pharmacologic screens or synthetic lethal genetic screens. Compounds found to only kill *NF1-*deficient cells could be then prioritized for further preclinical testing, using in vivo models of MPNST for example. Novel genetic targets identified from genome-wide synthetic lethal genetic screens could provide the basis for additional drug discovery efforts.

Efforts have been made to undertake this type of study for the *NF1* deficient cells. A small-scale screening project was conducted using mouse embryonic fibroblasts harboring biallelic loss of *NF1* [70]. This screening effort yielded only modest amounts of selective lethality toward *NF1* null cells and it is unclear how these could translate to the clinic. It would be most useful to undertake these types of screening efforts in cells of the correct cell-type for neurofibromas and MPNST (i.e., human Schwann cells). There are two cell-based platforms that would be amenable to the required genetic manipulation and required screening efforts: 1. Immortalized human Schwann cells. 2. iPSC derived Schwann cells. A number of immortalized human Schwann cell lines have been created that could be used for these types of assays [71]. These do have the limitation of being immortalized with retroviral vectors carrying *hTERT* for example and thus not mirroring the exact genetics of an MPNST cell. Induced pluripotent stem cell-based models are also a powerful tool for these types of studies. For example, protocols have been established to differentiate iPSC cells to the neural crest linage and then further to Schwann cells [64]. iPSC lines harboring *NF1* loss would have the advantage of being a “cleaner” genetic background, but perhaps not as easy to work with on large scale, genome-wide genetic screens. However, both systems could be used for: 1. Medium to large scale small molecule screens looking for drugs that could selectively kill *NF1* null cells. 2. Genome-wide genetic screens looking for other gene products whose loss would result in cell death when combined with *NF1* deficiency (Figure 2). The genetic screens are extremely powerful, as they offer a truly non-biased approach for novel target discovery. Whereas the small molecule screens could yield a potential therapeutic compound faster (particularity if the drug is already approved for another indication). 

### 3.5. Preclinical Development of New Therapies for NF1-Associated MPNST

A clear, standout success has been the identification of MEK inhibitors as effective treatment for symptomatic, inoperable plexiform neurofibromas [72]. Work examining the preclinical effectiveness of a selective pharmacological inhibitor of MEK, PD0325901, was reported in 2013 [12]. This work demonstrated that MAPK signaling suppression was effective in controlling neurofibroma growth in a neurofibromatosis mouse model (*Nf1^fl/fl^;Dhh-Cre*) and an NF1 patient MPNST cell xenograft. PD0325901 treatment increased survival of mice implanted with human MPNST cells, and shrank neurofibromas in more than 80% of the mice enrolled on treatment [73]. This important work clearly demonstrated that Ras/ERK signaling is critical for growth of NF1-associated PNSTs and provided reasoning to initiate clinical trials of MEK inhibitors for the treatment of these tumors in patients with NF1.

Following these promising results with PD0325901 in various in vivo models, investigation of other MEK inhibitors (including trametinib and selumetinib) were performed and advanced to human clinical trials. The most striking of these to date were the responses seen with selumetinib in pediatric patients with NF1 and inoperable plexiform neurofibromas [72]. Again, for this study the preclinical modeling of the MEK inhibitor was tested in the *Nf1^fl/fl^; Dhh-Cre* mouse model. As assessed by volumetric MRI, 67% of mice showed a reduction in tumor volume from baseline. When assessed in the human patient population, the results were marked, with confirmed partial responses in >70% of patients (≥20% decrease in volume from baseline). Moreover, disease progression was not reported in any patient and decreased tumor-related pain and functional impairment was widely reported. Recent Phase II trials also reported reduced tumor growth and in some cases a reduction in PN-associated pain and quality of life [74]. This is a major breakthrough in PN therapy and selumetinib was granted approval by the FDA for treatment of NF1-associated PN in April of 2020.

The success of selumetinib and other MEK inhibitors for NF1-associated PNs and other benign tumors represents a major milestone in therapy for this disease. However, several important questions remain. Selumetinib treatment effects are not complete, as tumors usually shrink by 20% or more, but do not disappear completely and typically regrow after treatment cessation [74]. It would be ideal if robust responses were observed in more patients. Moreover, MEK inhibitors have serious side effects when used long term, including skin rashes, ocular and cardiac toxicities [75]. Finally, it is unclear if MEK inhibition will be useful in the context of the premalignant atypical NF/ANNUBP in which *CDKN2A/2B* gene deletions are present or MPNSTs. 

Given the success of MEK inhibition for the control of plexiform neurofibromas and that many MPNST arise from within existing plexiform neurofibromas or ANNUBPs, it would seem likely that patients with MPNST could already be undergoing treatment with a MEK inhibitor. As such, it would be critical to establish any future targeted therapy for MPNST is at the least not antagonistic when used in combination with MEK inhibitors, such as selumetinib. Indeed, it would be desirable to identify novel candidate therapeutics exhibiting synergistic effects against MPNST models when used alongside MEK inhibitors.

### 3.6. Targeting Ras or Ras-Activated Signaling Pathways

Ras proteins are small, membrane associated GTPases that exist in an inactive guanosine diphosphate (GDP) bound form and an active guanosine triphosphate (GTP) bound form. Biallelic loss of the *NF1* gene, and the encoded protein neurofibromin, has been shown to lead to increased and prolonged Ras activation (i.e., Ras-GTP), including in benign and malignant peripheral nerve sheath tumors [10]. Ras-GTP itself has been considered undruggable as it lacks deep grooves that would fit a small molecule [76]. Ras proteins are post-translationally modified, including by farnesylation, and the enzymes that carry out these effects have been tested for therapeutic effects with some success in treating NF1-associated peripheral nerve sheath tumor cells [77,78,79]. However, translation into human clinical trials was disappointing [80,81]. Ras-GTP activated pathways have provided an alternate pathway to reverse the effects of *NF1* gene loss. A clear example, is Ras activation of the MAPK pathway, which proceeds from RAS activation to activation of a series of kinases: RAF to MEK to ERK(MAPK) kinase activation. MAPK ultimately phosphorylates many other substrates that can confer a survival or proliferation advantage to RAS activated cells [13]. As described above, a number of MEK inhibitors, notably selumetinib, have shown some clinical activity versus PN and other benign NF1-associated tumors [74,82]. 

### 3.7. Combination Signal Pathway Inhibition 

Given the incomplete effects of MEK inhibition alone on PN and MPNST, combinations with MEK inhibitors have been explored. MEK inhibitors have been shown to cooperate in vitro and in vivo with inhibition of the MNK kinases, which are active in many MPNSTs and converge on mTOR-dependent eIF4E phosphorylation [53]. Indeed, several papers show that *NF1*-deficient cells are relatively dependent on mTOR signaling and that mTORC1 inhibition synergizes with MEK inhibition [50]. mTORC1 inhibition as a single agent was weakly effective for PN treatment in patients [83]. Better results may be obtained with a direct mTOR kinase inhibitor that inhibits both mTORC1 and mTORC2 [84]. MEK inhibition and mTORC1, or mTORC1 and mTORC2 inhibition are synergistic in vitro and in vivo [13,84], and such a combination may be useful for PNs and MPNST treatment. The mTORC1 inhibitor sirolimus plus selumetinib is currently in a Phase II MPNST trial [NCT03433183; https://www.ctf.org/research/clinical-drug-pipeline]. mTOR inhibition was also combined with other signaling inhibitors including the HSP90 inhibitor ganetespib [NCT02008877], which did not produce responses in MPNST patients [85], despite promising preclinical data in the *NPCis* GEM model [86]. The mTORC1 inhibitor everolimus plus the VEGF inhibitor bevacizumab also failed to show potential in a Phase II MPNST study [87]. The multi-kinase inhibitor PLX3397 in combination with rapamycin inhibited MPNST xenografts in vivo [88], and this has led to a Phase II clinical trial [NCT02584647]. A more recent study revealed that blockade of mTOR with sapanisertib, which inhibits mTORC1 and mTORC2, with histone deacetylase (HDAC) inhibition is selectively toxic to Ras pathway-driven tumors, including human MPNST xenografts and the *NPCis* GEM model, by converging on the TXNIP/thioredoxin pathway [52]. The polo-like kinases (PLKs) have also emerged as targets for MPSNT therapy [89].

### 3.8. Targeting Cyclin-Dependent Kinases for MPNST

Premalignant atypical PN/ANNUBP have often acquired loss of *CDKN2A* and the *CDKN2B* genes, also a feature of many MPNSTs. These genes encode cyclin dependent kinase inhibitors and their loss is thought to lead to unregulated cyclin/cyclin dependent kinase (CDK) activity, in particular CDK4 or CDK6. Thus, these tumors may become sensitive to CDK4/6 inhibitors like ribociclib or palbociclib, which are approved for breast cancer treatment. Interestingly, high level expression of RABL6A in some MPNSTs promotes growth, in part by inhibition of RB1 protein, and its effects can be blocked by CDK4/6 inhibition [90]. CDK4/6 inhibition using ribociclib plus doxorubicin is in Phase II trial for MPNST now [NCT03009201].

### 3.9. Sensitivities Associated with Loss of PRC2 Function in MPNST Cells

As described above, progression to MPNST usually involves biallelic loss of the *EED* or *SUZ12* genes, essential components of the polycomb repressor complex 2 (PRC2). Most MPNSTs lack, or have reduced, histone H3 lysine 27 di- and tri-methylation, a chromatin repression mark mediated by EZH2, the enzymatic component of the PRC2 complex [22,24]. Loss of PRC2 activity has been proposed to sensitize MPNST cells to BRD4 inhibition, as BRD4 seems to be involved in activation of Ras pathway-dependent transcription dependent on histone H3 lysine 27 acetylation, which is enhanced following loss of methylation at this same site [25]. Another recent publication strongly suggests that loss of PRC2 in MPNST cells sensitizes them to histone deacetylase (HDAC) and DNA methyltransferase inhibition [91]. If these results are validated in isogenic model systems and proper in vivo preclinical MPNST models, these drugs should be tested in vivo, possibly with the signaling inhibitors described above that leverage sensitivities from loss of *NF1* expression. 

### 3.10. Other Therapeutic Approaches

Several other therapeutic approaches have made it to clinical trials for MPNST in recent years. Excitement for immunotherapies, especially checkpoint blockade and chimeric antigen receptor (CAR) T cell or CAR-T therapy has been building for treatment of MPNST. Indeed, blockade of PD1 [NCT02691026] or PD-1 and CTLA-4 [NCT02834013] are in clinical trials for MPNST. CAR-T cells specific for human EGFR are also being tested clinically in EGFR+ MPSNT [NCT03618381]. The perinuclear compartment disrupter metarrestin [92] is in Phase I trials for MPNST [NCT04222413], although not published specifically in MPNST. Inhibition of NFκB signaling and MPNST preclinical effectiveness was demonstrated using selinexor, a compound that induces IkB nuclear localization [93], and this agent is in Phase I testing for MPNST [NCT03880123]. Oncolytic viral therapy is a relatively new approach for MPNST therapy, but several reports describe promising preclinical data [94,95,96,97,98,99,100].

### 3.11. Comprehensive Pharmacological Profiling of Neurofibromatosis Type 1 Cancer Cell Lines

One attractive method for discovering new therapeutic options for NF1-associated tumors is to perform drug screens using one or more of the model systems described above. Drug screens in animal models are likely to be most predictive of future efficacy in vivo. Indeed, some measure of predictability for drugs to treat PNs in people was obtained using the *P0-Cre; Nf1^fl^/Nf1^fl^* or *Dhh-Cre.; Nf1^fl^/Nf1^fl^* models [12,87,101]. Unfortunately, these in vivo models are not well suited to testing many drugs and drug combinations. Therefore, screens using cell lines or cell strains have been used instead. In one medium throughput drug screen of about 470 compounds, immortalized *Nf1*-deficient mouse embryo fibroblasts (MEFs) were screened, using wildtype MEFs as a counter screen [70]. This project revealed several drugs with some selectivity for Nf1-deficienct cells. Moreover, validation of screening results using a human NF1-associated MPNST cell line xenograft model showed that nifedipine treatment significantly decreased local tumor growth. The reported data suggest that inhibitors of PP2A, including cantharidins, as well as calcium channel blockers like nifedipine, might be useful in MPNST treatment. A screen of this nature would benefit from the use of human isogenic cells of the correct cell lineage rather than fibroblasts. It is also possible that screening a larger number of drugs would yield additional drug candidates.

### 3.12. Synthetic Sensitivities Identified by Drug Screening in Nf1-Deficient Mouse Embryo Fibroblasts

In another in vitro drug screening effort, seven NF1-associated human MPNST and one sporadic MPNST cell line were used in a medium throughput screen of 130 drugs predicted to be useful for NF1-associated cancer therapy based on an analysis of the literature [102]. These drugs were based on mechanistic knowledge of neurofibromin and merlin function, as *NF2*-mutant cells were screened also, as well as important cancer pathways and classic chemotherapies. Drugs were found that clearly differentiated *NF1* from *NF2* mutant cells. Raf, MEK, PI3K/mTOR and Pak inhibitors were active versus *NF1*-mutant MPNST cells, while EGFR, GSK3, and AKT inhibitors had almost no activity. These data are consistent with the concept of using combination therapy centered on targeting the RAS-MAPK pathway in NF1-associated tumor cells. Additional drugs can then be layered on to maximize tumor-specific killing by targeting additional parallel vulnerabilities. This concept should be tested in vivo in improved models for NF1-associated peripheral nerve sheath tumors.

## 4. Summary and Future Perspectives

It has become clear that improved model systems are needed both to better validate current ideas before clinical testing in patients and to discover new MPNST treatment approaches and vulnerabilities. Too many MPNST clinical approaches have stalled at Phase II because they did not meet primary response rate endpoints and/or the effects were modest. The field is in desperate need of a home run. MPNST lack obvious activated oncogene products to target. Therefore, we favor the concept of using carefully engineered isogenic human Schwann lineage cell lines, harboring MPNST-relevant mutations, to screen for mutation-specific drug sensitivities and synthetic lethal genetic interactions to discover new therapies. Human iPSC derived model systems are likely to be especially useful in this regard. New therapeutic drugs and combinations must be better vetted before clinical testing and we favor testing in multiple human MPSNT PDXs and looking for evidence of very profound and long-lasting tumor shrinkage. Because in NF1 we can consider measures to reduce the chance of malignant progression we encourage the development of genetically accurate models in which benign PN, premalignant atypical PN, and MPNST can be studied using human cells and GEMMs. Immunoproficient GEMMs may also play an important role in development of new immune based and oncolytic viral therapies. Many good candidate therapeutic targets for *NF1*, *CDKN2A* and PRC2-deficient MPNST have emerged in recent years. These drugs, perhaps in combination, may also be relevant in tumors that characterize other RASopathies or which show activation of RAS-MAPK signaling after NF1 gene loss in sporadic settings.

## References

[1] Gutmann D.H., Ferner R.E., Listernick R.H., Korf B.R., Wolters P.L., Johnson K.J. (2017). Neurofibromatosis type 1. Nat. Rev. Dis Primers..

[2] Hernandez-Martin A., Duat-Rodriguez A. (2016). An Update on Neurofibromatosis Type 1: Not Just Cafe-au-Lait Spots and Freckling. Part II. Other Skin Manifestations Characteristic of NF1. NF1 and Cancer. Actas Dermosifiliogr..

[3] Longo J.F., Weber S.M., Turner-Ivey B.P., Carroll S.L. (2018). Recent Advances in the Diagnosis and Pathogenesis of Neurofibromatosis Type 1 (NF1)-associated Peripheral Nervous System Neoplasms. Adv. Anat. Pathol..

[4] Brosseau J.P., Liao C.P., Wang Y., Ramani V., Vandergriff T., Lee M., Patel A., Ariizumi K., Le L.Q. (2018). NF1 heterozygosity fosters de novo tumorigenesis but impairs malignant transformation. Nat. Commun..

[5] Miettinen M.M., Antonescu C.R., Fletcher C.D.M., Kim A., Lazar A.J., Quezado M.M., Reilly K.M., Stemmer-Rachamimov A., Stewart D.R., Viskochil D. (2017). Histopathologic evaluation of atypical neurofibromatous tumors and their transformation into malignant peripheral nerve sheath tumor in patients with neurofibromatosis 1-a consensus overview. Hum. Pathol..

[6] Beert E., Brems H., Daniels B., De Wever I., Van Calenbergh F., Schoenaers J., Debiec-Rychter M., Gevaert O., De Raedt T., Van Den Bruel A. (2011). Atypical neurofibromas in neurofibromatosis type 1 are premalignant tumors. Genes Chromosomes Cancer.

[7] Carrio M., Gel B., Terribas E., Zucchiatti A.C., Moline T., Rosas I., Teule A., Ramon Y.C.S., Lopez-Gutierrez J.C., Blanco I. (2018). Analysis of intratumor heterogeneity in Neurofibromatosis type 1 plexiform neurofibromas and neurofibromas with atypical features: Correlating histological and genomic findings. Hum. Mutat..

[8] Farid M., Demicco E.G., Garcia R., Ahn L., Merola P.R., Cioffi A., Maki R.G. (2014). Malignant peripheral nerve sheath tumors. Oncologist.

[9] Kolberg M., Holand M., Agesen T.H., Brekke H.R., Liestol K., Hall K.S., Mertens F., Picci P., Smeland S., Lothe R.A. (2013). Survival meta-analyses for >1800 malignant peripheral nerve sheath tumor patients with and without neurofibromatosis type 1. Neuro. Oncol..

[10] Ratner N., Miller S.J. (2015). A RASopathy gene commonly mutated in cancer: The neurofibromatosis type 1 tumour suppressor. Nat. Rev. Cancer.

[11] Laycock-van Spyk S., Thomas N., Cooper D.N., Upadhyaya M. (2011). Neurofibromatosis type 1-associated tumours: Their somatic mutational spectrum and pathogenesis. Hum. Genom..

[12] Jessen W.J., Miller S.J., Jousma E., Wu J., Rizvi T.A., Brundage M.E., Eaves D., Widemann B., Kim M.O., Dombi E. (2013). MEK inhibition exhibits efficacy in human and mouse neurofibromatosis tumors. J. Clin. Invest..

[13] Watson A.L., Anderson L.K., Greeley A.D., Keng V.W., Rahrmann E.P., Halfond A.L., Powell N.M., Collins M.H., Rizvi T., Moertel C.L. (2014). Co-targeting the MAPK and PI3K/AKT/mTOR pathways in two genetically engineered mouse models of schwann cell tumors reduces tumor grade and multiplicity. Oncotarget.

[14] Fischer-Huchzermeyer S., Chikobava L., Stahn V., Zangarini M., Berry P., Veal G.J., Senner V., Mautner V.F., Harder A. (2018). Testing ATRA and MEK inhibitor PD0325901 effectiveness in a nude mouse model for human MPNST xenografts. BMC Res. Notes..

[15] Peacock J.D., Cherba D., Kampfschulte K., Smith M.K., Monks N.R., Webb C.P., Steensma M. (2013). Molecular-guided therapy predictions reveal drug resistance phenotypes and treatment alternatives in malignant peripheral nerve sheath tumors. J. Transl. Med..

[16] Peacock J.D., Pridgeon M.G., Tovar E.A., Essenburg C.J., Bowman M., Madaj Z., Koeman J., Boguslawski E.A., Grit J., Dodd R.D. (2018). Genomic Status of MET Potentiates Sensitivity to MET and MEK Inhibition in NF1-Related Malignant Peripheral Nerve Sheath Tumors. Cancer Res..

[17] Ahsan S., Ge Y., Tainsky M.A. (2016). Combinatorial therapeutic targeting of BMP2 and MEK-ERK pathways in NF1-associated malignant peripheral nerve sheath tumors. Oncotarget..

[18] Verdijk R.M., den Bakker M.A., Dubbink H.J., Hop W.C., Dinjens W.N., Kros J.M. (2010). TP53 mutation analysis of malignant peripheral nerve sheath tumors. J. Neuropathol. Exp. Neurol..

[19] Brohl A.S., Kahen E., Yoder S.J., Teer J.K., Reed D.R. (2017). The genomic landscape of malignant peripheral nerve sheath tumors: Diverse drivers of Ras pathway activation. Sci. Rep..

[20] Kaplan H.G., Rostad S., Ross J.S., Ali S.M., Millis S.Z. (2018). Genomic Profiling in Patients With Malignant Peripheral Nerve Sheath Tumors Reveals Multiple Pathways With Targetable Mutations. J. Natl. Compr. Cancer Netw..

[21] Zhang M., Wang Y., Jones S., Sausen M., McMahon K., Sharma R., Wang Q., Belzberg A.J., Chaichana K., Gallia G.L. (2014). Somatic mutations of SUZ12 in malignant peripheral nerve sheath tumors. Nat. Genet..

[22] Lee W., Teckie S., Wiesner T., Ran L., Prieto Granada C.N., Lin M., Zhu S., Cao Z., Liang Y., Sboner A. (2014). PRC2 is recurrently inactivated through EED or SUZ12 loss in malignant peripheral nerve sheath tumors. Nat. Genet..

[23] Moritz L.E., Trievel R.C. (2018). Structure, mechanism, and regulation of polycomb repressive complex 2. J. Biol. Chem..

[24] Cleven A.H., Sannaa G.A., Briaire-de Bruijn I., Ingram D.R., van de Rijn M., Rubin B.P., de Vries M.W., Watson K.L., Torres K.E., Wang W.L. (2016). Loss of H3K27 tri-methylation is a diagnostic marker for malignant peripheral nerve sheath tumors and an indicator for an inferior survival. Mod. Pathol..

[25] De Raedt T., Beert E., Pasmant E., Luscan A., Brems H., Ortonne N., Helin K., Hornick J.L., Mautner V., Kehrer-Sawatzki H. (2014). PRC2 loss amplifies Ras-driven transcription and confers sensitivity to BRD4-based therapies. Nature.

[26] He S., Mansour M.R., Zimmerman M.W., Ki D.H., Layden H.M., Akahane K., Gjini E., de Groh E.D., Perez-Atayde A.R., Zhu S. (2016). Synergy between loss of NF1 and overexpression of MYCN in neuroblastoma is mediated by the GAP-related domain. Elife.

[27] Ki D.H., He S., Rodig S., Look A.T. (2017). Overexpression of PDGFRA cooperates with loss of NF1 and p53 to accelerate the molecular pathogenesis of malignant peripheral nerve sheath tumors. Oncogene.

[28] Ki D.H., Oppel F., Durbin A.D., Look A.T. (2019). Mechanisms underlying synergy between DNA topoisomerase I-targeted drugs and mTOR kinase inhibitors in NF1-associated malignant peripheral nerve sheath tumors. Oncogene.

[29] Oppel F., Tao T., Shi H., Ross K.N., Zimmerman M.W., He S., Tong G., Aster J.C., Look A.T. (2019). Loss of atrx cooperates with p53-deficiency to promote the development of sarcomas and other malignancies. PLoS Genet..

[30] Durbin A.D., Ki D.H., He S., Look A.T. (2016). Malignant Peripheral Nerve Sheath Tumors. Adv. Exp. Med. Biol..

[31] Gregorian C., Nakashima J., Dry S.M., Nghiemphu P.L., Smith K.B., Ao Y., Dang J., Lawson G., Mellinghoff I.K., Mischel P.S. (2009). PTEN dosage is essential for neurofibroma development and malignant transformation. Proc. Natl. Acad. Sci. USA.

[32] Keng V.W., Watson A.L., Rahrmann E.P., Li H., Tschida B.R., Moriarity B.S., Choi K., Rizvi T.A., Collins M.H., Wallace M.R. (2012). Conditional Inactivation of Pten with EGFR Overexpression in Schwann Cells Models Sporadic MPNST. Sarcoma.

[33] Huijbregts R.P., Roth K.A., Schmidt R.E., Carroll S.L. (2003). Hypertrophic neuropathies and malignant peripheral nerve sheath tumors in transgenic mice overexpressing glial growth factor beta3 in myelinating Schwann cells. J. Neurosci..

[34] Kazmi S.J., Byer S.J., Eckert J.M., Turk A.N., Huijbregts R.P., Brossier N.M., Grizzle W.E., Mikhail F.M., Roth K.A., Carroll S.L. (2013). Transgenic mice overexpressing neuregulin-1 model neurofibroma-malignant peripheral nerve sheath tumor progression and implicate specific chromosomal copy number variations in tumorigenesis. Am. J. Pathol..

[35] Brosius S.N., Turk A.N., Byer S.J., Longo J.F., Kappes J.C., Roth K.A., Carroll S.L. (2014). Combinatorial therapy with tamoxifen and trifluoperazine effectively inhibits malignant peripheral nerve sheath tumor growth by targeting complementary signaling cascades. J. Neuropathol. Exp. Neurol..

[36] Ling B.C., Wu J., Miller S.J., Monk K.R., Shamekh R., Rizvi T.A., Decourten-Myers G., Vogel K.S., DeClue J.E., Ratner N. (2005). Role for the epidermal growth factor receptor in neurofibromatosis-related peripheral nerve tumorigenesis. Cancer Cell..

[37] Rahrmann E.P., Moriarity B.S., Otto G.M., Watson A.L., Choi K., Collins M.H., Wallace M., Webber B.R., Forster C.L., Rizzardi A.E. (2014). Trp53 haploinsufficiency modifies EGFR-driven peripheral nerve sheath tumorigenesis. Am. J. Pathol..

[38] Keng V.W., Rahrmann E.P., Watson A.L., Tschida B.R., Moertel C.L., Jessen W.J., Rizvi T.A., Collins M.H., Ratner N., Largaespada D.A. (2012). PTEN and NF1 Inactivation in Schwann Cells Produces a Severe Phenotype in the Peripheral Nervous System That Promotes the Development and Malignant Progression of Peripheral Nerve Sheath Tumors. Cancer Res..

[39] Mo W., Chen J., Patel A., Zhang L., Chau V., Li Y., Cho W., Lim K., Xu J., Lazar A.J. (2013). CXCR4/CXCL12 mediate autocrine cell- cycle progression in NF1-associated malignant peripheral nerve sheath tumors. Cell.

[40] Johannessen C.M., Johnson B.W., Williams S.M., Chan A.W., Reczek E.E., Lynch R.C., Rioth M.J., McClatchey A., Ryeom S., Cichowski K. (2008). TORC1 is essential for NF1-associated malignancies. Curr. Biol..

[41] Rhodes S.D., He Y., Smith A., Jiang L., Lu Q., Mund J., Li X., Bessler W., Qian S., Dyer W. (2019). Cdkn2a (Arf) loss drives NF1-associated atypical neurofibroma and malignant transformation. Hum. Mol. Genet..

[42] Paez-Ribes M., Gonzalez-Gualda E., Doherty G.J., Munoz-Espin D. (2019). Targeting senescent cells in translational medicine. EMBO Mol. Med..

[43] Rahrmann E.P., Watson A.L., Keng V.W., Choi K., Moriarity B.S., Beckmann D.A., Wolf N.K., Sarver A., Collins M.H., Moertel C.L. (2013). Forward genetic screen for malignant peripheral nerve sheath tumor formation identifies new genes and pathways driving tumorigenesis. Nat. Genet..

[44] Wu J., Keng V.W., Patmore D.M., Kendall J.J., Patel A.V., Jousma E., Jessen W.J., Choi K., Tschida B.R., Silverstein K.A. (2016). Insertional Mutagenesis Identifies a STAT3/Arid1b/beta-catenin Pathway Driving Neurofibroma Initiation. Cell Rep..

[45] Laugesen A., Hojfeldt J.W., Helin K. (2019). Molecular Mechanisms Directing PRC2 Recruitment and H3K27 Methylation. Mol. Cell.

[46] Streubel G., Watson A., Jammula S.G., Scelfo A., Fitzpatrick D.J., Oliviero G., McCole R., Conway E., Glancy E., Negri G.L. (2018). The H3K36me2 Methyltransferase Nsd1 Demarcates PRC2-Mediated H3K27me2 and H3K27me3 Domains in Embryonic Stem Cells. Mol. Cell.

[47] Vogel K.S., Klesse L.J., Velasco-Miguel S., Meyers K., Rushing E.J., Parada L.F. (1999). Mouse tumor model for neurofibromatosis type 1. Science.

[48] Cichowski K., Shih T.S., Schmitt E., Santiago S., Reilly K., McLaughlin M.E., Bronson R.T., Jacks T. (1999). Mouse models of tumor development in neurofibromatosis type 1. Science.

[49] Reilly K.M., Tuskan R.G., Christy E., Loisel D.A., Ledger J., Bronson R.T., Smith C.D., Tsang S., Munroe D.J., Jacks T. (2004). Susceptibility to astrocytoma in mice mutant for Nf1 and Trp53 is linked to chromosome 11 and subject to epigenetic effects. Proc. Natl. Acad. Sci. USA.

[50] Malone C.F., Fromm J.A., Maertens O., DeRaedt T., Ingraham R., Cichowski K. (2014). Defining key signaling nodes and therapeutic biomarkers in NF1-mutant cancers. Cancer Discov..

[51] Maertens O., McCurrach M.E., Braun B.S., De Raedt T., Epstein I., Huang T.Q., Lauchle J.O., Lee H., Wu J., Cripe T.P. (2017). A Collaborative Model for Accelerating the Discovery and Translation of Cancer Therapies. Cancer Res..

[52] Malone C.F., Emerson C., Ingraham R., Barbosa W., Guerra S., Yoon H., Liu L.L., Michor F., Haigis M., Macleod K.F. (2017). mTOR and HDAC Inhibitors Converge on the TXNIP/Thioredoxin Pathway to Cause Catastrophic Oxidative Stress and Regression of RAS-Driven Tumors. Cancer Discov..

[53] Lock R., Ingraham R., Maertens O., Miller A.L., Weledji N., Legius E., Konicek B.M., Yan S.C., Graff J.R., Cichowski K. (2016). Cotargeting MNK and MEK kinases induces the regression of NF1-mutant cancers. J. Clin. Invest..

[54] Dodd R.D., Mito J.K., Eward W.C., Chitalia R., Sachdeva M., Ma Y., Barretina J., Dodd L., Kirsch D.G. (2013). NF1 deletion generates multiple subtypes of soft-tissue sarcoma that respond to MEK inhibition. Mol. Cancer.

[55] Dodd R.D., Lee C.L., Overton T., Huang W., Eward W.C., Luo L., Ma Y., Ingram D.R., Torres K.E., Cardona D.M. (2017). NF1(+/−) Hematopoietic Cells Accelerate Malignant Peripheral Nerve Sheath Tumor Development without Altering Chemotherapy Response. Cancer Res..

[56] Huang J., Chen M., Whitley M.J., Kuo H.C., Xu E.S., Walens A., Mowery Y.M., Van Mater D., Eward W.C., Cardona D.M. (2017). Generation and comparison of CRISPR-Cas9 and Cre-mediated genetically engineered mouse models of sarcoma. Nat. Commun..

[57] Fang Y., Elahi A., Denley R.C., Rao P.H., Brennan M.F., Jhanwar S.C. (2009). Molecular characterization of permanent cell lines from primary, metastatic and recurrent malignant peripheral nerve sheath tumors (MPNST) with underlying neurofibromatosis-1. Anticancer Res..

[58] Sun D., Tainsky M.A., Haddad R. (2012). Oncogene Mutation Survey in MPNST Cell Lines Enhances the Dominant Role of Hyperactive Ras in NF1 Associated Pro-Survival and Malignancy. Transl Oncogenomics.

[59] Castellsague J., Gel B., Fernandez-Rodriguez J., Llatjos R., Blanco I., Benavente Y., Perez-Sidelnikova D., Garcia-Del Muro J., Vinals J.M., Vidal A. (2015). Comprehensive establishment and characterization of orthoxenograft mouse models of malignant peripheral nerve sheath tumors for personalized medicine. Embo Mol. Med..

[60] Pollard K., Banerjee J., Doan X., Wang J., Guo X., Allaway R., Langmead S., Slobogean B., Meyer C.F., Loeb D.M. A clinically and genomically annotated nerve sheath tumor biospecimen repository. biorxiv.

[61] Rutkowski J.L., Kirk C.J., Lerner M.A., Tennekoon G.I. (1995). Purification and expansion of human Schwann cells in vitro. Nat. Med..

[62] Li H., Chang L.J., Neubauer D.R., Muir D.F., Wallace M.R. (2016). Immortalization of human normal and NF1 neurofibroma Schwann cells. Lab. Invest..

[63] Watson A.L., Rahrmann E.P., Moriarity B.S., Choi K., Conboy C.B., Greeley A.D., Halfond A.L., Anderson L.K., Wahl B.R., Keng V.W. (2013). Canonical Wnt/beta-catenin signaling drives human schwann cell transformation, progression, and tumor maintenance. Cancer Discov..

[64] Carrio M., Mazuelas H., Richaud-Patin Y., Gel B., Terribas E., Rosas I., Jimenez-Delgado S., Biayna J., Vendredy L., Blanco I. (2019). Reprogramming Captures the Genetic and Tumorigenic Properties of Neurofibromatosis Type 1 Plexiform Neurofibromas. Stem. Cell Rep..

[65] Dobzhansky T. (1946). Genetics of natural populations; recombination and variability in populations of Drosophila pseudoobscura. Genetics.

[66] Hartwell L.H., Szankasi P., Roberts C.J., Murray A.W., Friend S.H. (1997). Integrating genetic approaches into the discovery of anticancer drugs. Science.

[67] Bryant H.E., Schultz N., Thomas H.D., Parker K.M., Flower D., Lopez E., Kyle S., Meuth M., Curtin N.J., Helleday T. (2005). Specific killing of BRCA2-deficient tumours with inhibitors of poly(ADP-ribose) polymerase. Nature.

[68] Javle M., Curtin N.J. (2011). The role of PARP in DNA repair and its therapeutic exploitation. Br. J. Cancer.

[69] Huang A., Garraway L.A., Ashworth A., Weber B. (2020). Synthetic lethality as an engine for cancer drug target discovery. Nat. Rev. Drug Discov..

[70] Semenova G., Stepanova D.S., Deyev S.M., Chernoff J. (2017). Medium throughput biochemical compound screening identifies novel agents for pharmacotherapy of neurofibromatosis type 1. Biochimie.

[71] Ferrer M., Gosline S.J.C., Stathis M., Zhang X., Guo X., Guha R., Ryman D.A., Wallace M.R., Kasch-Semenza L., Hao H. (2018). Pharmacological and genomic profiling of neurofibromatosis type 1 plexiform neurofibroma-derived schwann cells. Sci. Data.

[72] Dombi E., Baldwin A., Marcus L.J., Fisher M.J., Weiss B., Kim A., Whitcomb P., Martin S., Aschbacher-Smith L.E., Rizvi T.A. (2016). Activity of Selumetinib in Neurofibromatosis Type 1-Related Plexiform Neurofibromas. N. Engl. J. Med..

[73] McCowage G.B., Pratilas C.A., Hargrave D.R., Moertel C.L., Whitlock J., Fox E., Hingorani P., Russo M.W., Dasgupta K., Tseng L. (2018). Trametinib in pediatric patients with neurofibromatosis type 1 (NF-1)–associated plexiform neurofibroma: A phase I/IIa study. J. Clin. Oncol..

[74] Gross A.M., Wolters P.L., Dombi E., Baldwin A., Whitcomb P., Fisher M.J., Weiss B., Kim A., Bornhorst M., Shah A.C. (2020). Selumetinib in Children with Inoperable Plexiform Neurofibromas. N. Engl. J. Med..

[75] Daud A., Tsai K. (2017). Management of Treatment-Related Adverse Events with Agents Targeting the MAPK Pathway in Patients with Metastatic Melanoma. Oncologist.

[76] Stalnecker C.A., Der C.J. (2020). RAS, wanted dead or alive: Advances in targeting RAS mutant cancers. Sci. Signal..

[77] Kim H.A., Ling B., Ratner N. (1997). Nf1-deficient mouse Schwann cells are angiogenic and invasive and can be induced to hyperproliferate: Reversion of some phenotypes by an inhibitor of farnesyl protein transferase. Mol. Cell Biol..

[78] Dilworth J.T., Wojtkowiak J.W., Mathieu P., Tainsky M.A., Reiners J.J., Mattingly R.R., Hancock C.N. (2008). Suppression of proliferation of two independent NF1 malignant peripheral nerve sheath tumor cell lines by the pan-ErbB inhibitor CI-1033. Cancer Biol..

[79] Wojtkowiak J.W., Fouad F., LaLonde D.T., Kleinman M.D., Gibbs R.A., Reiners J.J., Borch R.F., Mattingly R.R. (2008). Induction of apoptosis in neurofibromatosis type 1 malignant peripheral nerve sheath tumor cell lines by a combination of novel farnesyl transferase inhibitors and lovastatin. J. Pharm. Exp..

[80] Widemann B.C., Salzer W.L., Arceci R.J., Blaney S.M., Fox E., End D., Gillespie A., Whitcomb P., Palumbo J.S., Pitney A. (2006). Phase I trial and pharmacokinetic study of the farnesyltransferase inhibitor tipifarnib in children with refractory solid tumors or neurofibromatosis type I and plexiform neurofibromas. J. Clin. Oncol..

[81] Widemann B.C., Dombi E., Gillespie A., Wolters P.L., Belasco J., Goldman S., Korf B.R., Solomon J., Martin S., Salzer W. (2014). Phase 2 randomized, flexible crossover, double-blinded, placebo-controlled trial of the farnesyltransferase inhibitor tipifarnib in children and young adults with neurofibromatosis type 1 and progressive plexiform neurofibromas. Neuro. Oncol..

[82] Fangusaro J., Onar-Thomas A., Young Poussaint T., Wu S., Ligon A.H., Lindeman N., Banerjee A., Packer R.J., Kilburn L.B., Goldman S. (2019). Selumetinib in paediatric patients with BRAF-aberrant or neurofibromatosis type 1-associated recurrent, refractory, or progressive low-grade glioma: A multicentre, phase 2 trial. Lancet. Oncol..

[83] Weiss B., Widemann B.C., Wolters P., Dombi E., Vinks A., Cantor A., Perentesis J., Schorry E., Ullrich N., Gutmann D.H. (2015). Sirolimus for progressive neurofibromatosis type 1-associated plexiform neurofibromas: A neurofibromatosis Clinical Trials Consortium phase II study. Neuro. Oncol..

[84] Varin J., Poulain L., Hivelin M., Nusbaum P., Hubas A., Laurendeau I., Lantieri L., Wolkenstein P., Vidaud M., Pasmant E. (2016). Dual mTORC1/2 inhibition induces anti-proliferative effect in NF1-associated plexiform neurofibroma and malignant peripheral nerve sheath tumor cells. Oncotarget.

[85] Kim A., Lu Y., Okuno S.H., Reinke D., Maertens O., Perentesis J., Basu M., Wolters P.L., De Raedt T., Chawla S. (2020). Targeting Refractory Sarcomas and Malignant Peripheral Nerve Sheath Tumors in a Phase I/II Study of Sirolimus in Combination with Ganetespib (SARC023). Sarcoma.

[86] De Raedt T., Walton Z., Yecies J.L., Li D., Chen Y., Malone C.F., Maertens O., Jeong S.M., Bronson R.T., Lebleu V. (2011). Exploiting cancer cell vulnerabilities to develop a combination therapy for ras-driven tumors. Cancer Cell.

[87] Widemann B.C., Lu Y., Reinke D., Okuno S.H., Meyer C.F., Cote G.M., Chugh R., Milhem M.M., Hirbe A.C., Kim A. (2019). Targeting Sporadic and Neurofibromatosis Type 1 (NF1) Related Refractory Malignant Peripheral Nerve Sheath Tumors (MPNST) in a Phase II Study of Everolimus in Combination with Bevacizumab (SARC016). Sarcoma.

[88] Patwardhan P.P., Surriga O., Beckman M.J., de Stanchina E., Dematteo R.P., Tap W.D., Schwartz G.K. (2014). Sustained inhibition of receptor tyrosine kinases and macrophage depletion by PLX3397 and rapamycin as a potential new approach for the treatment of MPNSTs. Clin. Cancer Res..

[89] Kolberg M., Bruun J., Murumagi A., Mpindi J.P., Bergsland C.H., Holand M., Eilertsen I.A., Danielsen S.A., Kallioniemi O., Lothe R.A. (2017). Drug sensitivity and resistance testing identifies PLK1 inhibitors and gemcitabine as potent drugs for malignant peripheral nerve sheath tumors. Mol. Oncol..

[90] Kohlmeyer J.L., Kaemmer C.A., Pulliam C., Maharjan C.K., Moreno Samayoa A., Major H.J., Cornick K.E., Knepper-Adrian V., Khanna R., Sieren J.C. RABL6A is an essential driver of MPNSTs that negatively regulates the RB1 pathway and sensitizes tumor cells to CDK4/6 inhibitors. Clin. Cancer Res..

[91] Wojcik J.B., Marchione D.M., Sidoli S., Djedid A., Lisby A., Majewski J., Garcia B.A. (2019). Epigenomic reordering induced by Polycomb loss drives oncogenesis but leads to therapeutic vulnerabilities in malignant peripheral nerve sheath tumors. Cancer Res..

[92] Frankowski K.J., Wang C., Patnaik S., Schoenen F.J., Southall N., Li D., Teper Y., Sun W., Kandela I., Hu D. (2018). Metarrestin, a perinucleolar compartment inhibitor, effectively suppresses metastasis. Sci. Transl. Med..

[93] Nair J.S., Musi E., Schwartz G.K. (2017). Selinexor (KPT-330) Induces Tumor Suppression through Nuclear Sequestration of IkappaB and Downregulation of Survivin. Clin. Cancer Res..

[94] Mahller Y.Y., Rangwala F., Ratner N., Cripe T.P. (2006). Malignant peripheral nerve sheath tumors with high and low Ras-GTP are permissive for oncolytic herpes simplex virus mutants. Pediatr Blood Cancer..

[95] Farassati F., Pan W., Yamoutpour F., Henke S., Piedra M., Frahm S., Al-Tawil S., Mangrum W.I., Parada L.F., Rabkin S.D. (2008). Ras signaling influences permissiveness of malignant peripheral nerve sheath tumor cells to oncolytic herpes. Am. J. Pathol..

[96] Antoszczyk S., Spyra M., Mautner V.F., Kurtz A., Stemmer-Rachamimov A.O., Martuza R.L., Rabkin S.D. (2014). Treatment of orthotopic malignant peripheral nerve sheath tumors with oncolytic herpes simplex virus. Neuro Oncol..

[97] Deyle D.R., Escobar D.Z., Peng K.W., Babovic-Vuksanovic D. (2015). Oncolytic measles virus as a novel therapy for malignant peripheral nerve sheath tumors. Genes.

[98] Jackson J.D., Markert J.M., Li L., Carroll S.L., Cassady K.A. (2016). STAT1 and NF-kappaB Inhibitors Diminish Basal Interferon-Stimulated Gene Expression and Improve the Productive Infection of Oncolytic HSV in MPNST Cells. Mol. Cancer Res..

[99] Currier M.A., Sprague L., Rizvi T.A., Nartker B., Chen C.Y., Wang P.Y., Hutzen B.J., Franczek M.R., Patel A.V., Chaney K.E. (2017). Aurora A kinase inhibition enhances oncolytic herpes virotherapy through cytotoxic synergy and innate cellular immune modulation. Oncotarget.

[100] Ghonime M.G., Cassady K.A. (2018). Combination Therapy Using Ruxolitinib and Oncolytic HSV Renders Resistant MPNSTs Susceptible to Virotherapy. Cancer Immunol. Res..

[101] Robertson K.A., Nalepa G., Yang F.C., Bowers D.C., Ho C.Y., Hutchins G.D., Croop J.M., Vik T.A., Denne S.C., Parada L.F. (2012). Imatinib mesylate for plexiform neurofibromas in patients with neurofibromatosis type 1: A phase 2 trial. Lancet. Oncol..

[102] Guo J., Grovola M.R., Xie H., Coggins G.E., Duggan P., Hasan R., Huang J., Lin D.W., Song C., Witek G.M. (2017). Comprehensive pharmacological profiling of neurofibromatosis cell lines. Am. J. Cancer Res..

